# Chicago Medical Society

**Published:** 1887-01

**Authors:** 


### CHICAGO MEDICAL SOCIETY.

Officially Reported for The Atlanta Medical and Surgical Journal.

Stated Meeting, Nov. 15, 1886.

The President, E. J. Doering, M. D., in the chair.

Dr. Lester Curtis read a paper on

THE ABSENCE OF THE PATELLA TENDON REFLEX, in which he enumerated a number of cases of persons in apparently good health in whom the patella tendon reflex cannot be elicited, such being the case with the author himself. He quoted from authors who had made similar observations, and who had also found the patella tendon reflex present in diseases in which it is not expected. These variations in the presence and absence of the patella tendon reflex were so frequent that he was inclined to question its absence as an important sign of nervous disease. He believed it to be a sign that had received undue attention.

Dr. D. R. Brower, in opening the discussion, said: I have been very much interested in Dr. Curtis's paper. I have found the patella tendon reflex occasionally absent in health, but very rarely. Since the suggestion was made by Jendrassik that an effort be made to increase the general muscular tension by having the person pull upon the fingers of one hand linked into those of the other, which was brought to my attention about a year ago, I have been able to elicit the patella reflex in healthy persons when otherwise I would have failed. I think it is necessary in doubtful cases that the blow be struck upon the naked limb. I think it is occasionally absent in health, and it has been my habit in teaching to always say in regard to the patella tendon reflex that it is the loss and not the absence ; we have first to know that the person had patella tendon reflex before we can regard its absence as pathological. I have taken that view of the matter for a long time. As to the presence of tendon reflex in locomotor ataxia, the President will remember one of the patients that I saw three or four years ago, in whom patella reflex was present and in whom all of the other symptoms of locomotor ataxia were present in a marked degree, and I have met with that condition in one or two other cases. I think that in these cases the degeneration has not commenced in the usual place. At the meeting of the American Medical Association an interesting paper on this subject was read by Dr. Zenner, of Cincinnati, who has made very extensive observations upon healthy people, as well as upon the insane, with a view of determining the real value of this sign. And his opinion is in accord with my own, that if all precautions are taken it is a remarkable thing for it to be absent in a healthy person. In the case cited by the author there would seem to be a part of the spinal cord that was not entirely destroyed, possibly enough to admit of the passage of the stimulus from the sensory nerves.

Dr. H. N. Moyer said regarding the manner of eliciting the patella tendon reflex: Unless very great care is exercised in determining that the reflex is absent the observation has very little value. In testing this reflex I prefer to have the leg rest across the back of the wrist, by which means we easily detect any tension of the hamstring muscles, which greatly interferes with the re-action. As to the manner in which the tendon should be struck, I do not believe that the surface of the ulnar side of the hand is the proper thing to elicit it, and the ordinary rubber hammer is not quite heavy enough for that purpose. The best thing is a steel hammer with a small rubber tip. In doubtful cases care should be taken to percuss the tendon over its entire surface. I have seen a number of cases in which I supposed the patella tendon reflex was absent in undoubtedly healthy persons, but since this new manner of increasing muscular tonicity by lessening the inhibitory power of the brain over the lower spinal centres has been adopted, have never seen a case in which the patella reflex was absent in a healthy individual. There is one other point in regard to the difference in the amount of the reflex obtained when this procedure is employed and when it is not. There are certain qualitative variations which have not been pointed out. I have under my observation at present two cases of corea in which there is only the slightest evidence of patella tendon reflex on percussion in the ordinary way, but when the patients are required to clasp the hands and the patella is then struck the reflex is markedly exaggerated. I think in some cases this procedure will enable us to diagnosticate between the neurasthenia of cerebral and of spinal origin. I have one undoubted case of locomotor ataxia in which the patella tendon reflex is present. In the two cases of Westphal which have been recently published, the microscope revealed the morbid processes in the lumbar portion of the cord to be largely confined to the points of entrance of the posterior roots, and also to a slight extent involving the lateral columns. This probably obtains in the cases reported to-night.

Dr. J. J. M. Angear said: I do not know that we can come to the conclusion that our patient is suffering from locomotor ataxia when we find the patella tendon reflex absent. I have in my mind a patient, who was under my care about two years ago, in whom the patella tendon reflex was entirely absent. There was unsteadiness in gait, a little wavy motion when attempting to stand erect, with eyes closed and feet close together, and the patient walked with a cane. There were symptoms of congestion of the cord, a feeling of a heavy weight at the feet, that peculiar sensation known as girdle feeling, which improved on lying down. The patient improved somewhat under the use of mercury and iodide of potash (I learned afterward that when a young man he had syphilis, but it has not shown itself since). This was two years ago, and the patient is no worse to-day, but it seems to me that he ought to be a great deal worse if it was locomotor ataxia. If we fail to elicit the reflex by simply striking the tendon, we can get it, if it is present, by engaging the patient in conversation about something foreign to himself and then suddenly strike the tendon. In regard to cases where it is absent in apparent health, I think we should emphasize *apparent*, for we have no positive evidence that the nervous system is in a normal condition in such cases, and I should be a little skeptical with regard to the patient's perfect health if I had taken all the precautions of securing this action and then found it absent.

Dr. Robert Tilley said: I wish to bear testimony to the truth of Dr. Curtis's observations. I certainly have seen a small number of persons that I considered average healthy individuals in whom I have not been able to elicit the patella tendon reflex. I have not caused the patients to bear the leg, but I have employed other devices to satisfy myself that if the tendon reflex was not absolutely absent it was certainly diminished in its activity. I would like to emphasize the fact that it seems under certain circumstances to be obtained when struck upon one side or the other when it cannot be obtained by striking the tendom in the centre. On several occasions it has struck me as desirable in post mortem examinations to have a microscopic examination of the tendon itself, and see if some peculiar changes might not be visible in it. I would ask Dr. Curtis if he knows of any histological investigations in that direction. I recently saw an article giving an account of a case where the tendon reflex seemed to be absent, but on the administration of a small dose of morphia it was elicited. No mention has been made of what may be called a normal tendon reflex, although the exaggerated form has been referred to, and I do not know whether any one here would venture to give a definition of a normal tendon reflex in opposition to an exaggerated one. I am certainly disposed to take exception to Dr. Curtis's explanation of the tendon reflex, that it is most likely to be found in individuals who are a little scary, are easily frightened, easily thrown off their guard, or persons of that character. I may instance myself. I do not think I should be easily thrown off my guard, but in my case the tendon reflex is markedly present, so much so that those who would distinguish between the forms would say that mine was an exaggerated form.

Dr. J. Frank said: I have a theory as to why the tendon reflex might be absent in a healthy person. In order to get the reflex the tendon must be put on the stretch, and there are such things as abnormal patella tendons. If the ligamentum patellæ is abnormally long when the leg is flexed, the tendon is not put on the stretch; if lax and cannot be made tense, it might be struck all day without any result. I think this will explain why in some people the tendon reflex is absent. When I was a student my preceptor tried it on me, but could not get any tendon reflex; it seems that at certain times my tendon answers to the blow and at other times it does not; still I have been in good health all my life. Where the tendon reflex is absent without other symptoms the tendon should be examined.

Dr. James Jewell said that in tendon reflex, when the muscle is struck a shock is given to the sense nerves. He had met with a large number of cases in which the patella tendon reflex could not be obtained by the ordinary impulse, but which he had been able to elicit by other means. He thought locomotor ataxia did not depend so much upon loss of sensibility in the muscles themselves as in the great numbness of the feet. He has had cases of locomotor ataxia in which the only symptom was acute sensibility to temperature, and another who had no sensibility on the right side except in the nates.

Dr. Joseph Zeisler read a paper on

THE USE OF ICHTHYOL IN THE TREATMENT OF SKIN DISEASES.

Dr. Zeisler gave his clinical experience with ichthyol in the treatment of over one hundred cases of skin disease. The strength of the salve varied from 3 to 30 per cent.; frequently other drugs were added as adjuvants according to the requirements of the case. All the usual ointment bases mix well with it. Ichthyol soap was found very useful for acne rosacea and sycosis. The physiological effects of ichthyol, its regenerative power when used in a mild form, its resolvent action when used in full strength, its contracting influence upon blood-vessels, were explained from its chief quality to draw oxygen from the tissues. In about twenty-five cases it was used internally in the form of capsules (0.10 pro dosi), three to ten of which may be taken daily. In this form Dr. Zeisler found it very useful in chronic cases, and thinks that it may frequently be preferable to arsenic. Very good results were obtained in eczema, fifty-six cases of which, comprising nearly all forms and stages of that disease, were treated with it; its effect and the mode of its application in the principal form is described and illustrated in the history of two cases.

In sycosis (eleven cases) and psoriasis (two cases), it was found to be a very good adjuvant. Excellent results were also obtained from internal and external use of ichthyol in acne rosacea (seventeen cases). In acne vulgaris (ten cases) it did not result beneficially, except when used internally. In several other cases, herpes tonsurans, prurigo and acne varialiformis, it was used in too few cases to allow of any decided conclusion.

Dr. G. C. Paoli said there is no one remedy that we can positively rely on in the treatment of skin diseases. This new remedy, ichthyol, was used by Dr. Unna, of Hamburg, but he combined it with other ointments. We cannot treat all the stages of skin diseases with the same remedy. Ichthyol has a fetid odor, and he should think it would be obnoxious in use. He has not had an opportunity to try it.

Dr. E. J. Kuh said: Not enough time has elapsed to express either a final condemnatory or laudatory opinion on ichthyol. The physiological experiments by Baumann and Schotten are entirely insufficient, and the conclusions reached are practically no conclusions at all. Our clinical knowledge has been sufficient to show its value in skin diseases ; and as Dr. Zeisler has confined himself to a discussion of its use in his specialty, a few words bearing on the application of ichthyol in other diseases may not be out of place. Lorenz and Unna are its chief recommenders. The former in the beginning applied it in acute and chronic articular rheumatism, and to judge from the experiences of others (including myself) who have used it in this direction, there can be no doubt that it is a very strong adjuvant in this disease, and that the exhibition of salicylates is promoted in its efficiency when used in conjunction with ichthyol salve. Lorenz extended its use to gout, muscular rheumatism, contusions, gastric troubles, etc. Unna, whose enthusiasm is so unbounded as to inspire distrust, barely finds a limit to its usefulness. It is a mild anti-tuberculosum, has cured asthma in an eczema patient, will cause immediate demarcation in erysipelas, etc. It is a malicious characteristic of new remedies that they are wonderfully multiple in their therapeutic action. Time, the great enemy of the pharmacopœia, is the surest test. Let us hope that it may deal gently with ichthyol.

Dr. Jewell thought a great deal of attention should be paid to the constitutional conditions, and especially to the neurotic aspect of cases of eczema. He had met with success in the treatment of skin diseases by a current of large quantity and mild intensity from an electric battery.

Dr. John A. Robinson said that the literature on the subject of ichthyol is very meagre, except what is found in foreign and special journals. He was sorry that Dr. Zeisler did not give a fuller description of the drug, in order that general practitioners might become more familiar with it. Undoubtedly many of the members present thought it a proprietary preparation, and therefore will not investigate its merits more closely. During the past year a large number of new remedies have been introduced which unfortunately bear a foreign patent, although the manner of manufacture is known. While opposed to prescribing secret proprietary preparations, he believed the therapeutical action of such drugs as ichthyol, antipyrin, thallin, etc., should be studied.

Dr. R. Tilley said that it was to be regretted that the author did not present some cases that were treated solely by ichthyol; for instance, the case that went to so many physicians, the salicylate given would be likely to exert quite as much influence as the ichthyol, and the same might be said of the case treated by hydrarg. ammoniatum. It is really difficult to form an estimate as to what we can expect from ichthyol by the result of the combinations which the author found it necessary to use.

Dr. Joseph Zeisler, in closing the discussion, said that he did not think of recommending ichthyol as a panacea; on the contrary, he was afraid that it would be over-estimated, and that some would use it in cases where it might not be beneficial. As to the remarks of Dr. Kuh, the physiological effect of ichthyol was not tested by experiments on animals, but was inferred from clinical observations. He was very much interested in Dr. Jewell's suggestion, and referred to the well-known forms of neurotic eczema.

Ichthyol has been found in Tyrol, Austria, in some bituminous rocks. He was unable to tell anything about its manufacture; Mr. Sargent gets it directly from Germany. In reply to Dr. Tilley, he said it was true that in some cases he could not say how much of the benefit was due to internal and how much to external treatment, how much to ichthyol and how much to other remedies. But his observations on ichthyol might still have some value when compared with other cases where *ceteris paribus* it is not used.

